# Association Between Serum Leptin Concentrations and Disease Severity Across Airway Disease Phenotypes: A Single-Center Retrospective Observational Study

**DOI:** 10.3390/diseases14080301

**Published:** 2026-08-19

**Authors:** Corina Porr, Valentin-Cristian Iovin, Anca Vidrighin, Emi Marinela Preda, Gabriela Mariana Iancu, Dana M. Harris, Cosmina Diaconu

**Affiliations:** 1 Allergology Department, Faculty of Medicine, Lucian Blaga University of Sibiu, 550169 Sibiu, Romania; corina.porr@ulbsibiu.ro; 2 Allergology Department, County Clinical Emergency Hospital Sibiu, Bulevardul Corneliu Coposu 2–4, 550245 Sibiu, Romania; 3 Doctoral School Department, Victor Babeș University of Medicine and Pharmacy of Timișoara, 300041 Timișoara, Romania; valentin.iovin@umft.ro; 4 Department III Functional Sciences, Physiology Discipline, Victor Babeș University of Medicine and Pharmacy of Timișoara, 300041 Timișoara, Romania; 5 Centre of Immuno-Physiology and Biotechnologies (CIFBIOTEH), Department of Functional Sciences, Physiology, Victor Babeș University of Medicine and Pharmacy of Timișoara, 300041 Timișoara, Romania; 6 Department of Paediatrics, Faculty of Medicine, Lucian Blaga University of Sibiu, 550169 Sibiu, Romania; 7 Department of Radiology, Carol Davila University of Medicine and Pharmacy, 050474 Bucharest, Romania; emi.preda@umfcd.ro; 8 Department of Radiology and Medical Imaging, Foișor Clinical Hospital of Orthopaedics, Traumatology and Osteoarticular TB, 021382 Bucharest, Romania; 9 Department of Dermatology, Faculty of Medicine, Lucian Blaga University of Sibiu, 550169 Sibiu, Romania; 10 Clinic of Dermatology, County Clinical Emergency Hospital Sibiu, Bulevardul Corneliu Coposu 2–4, 550245 Sibiu, Romania; 11 Department of Internal Medicine, Mayo Clinic, Jacksonville, FL 32224, USA; harris.dana@mayo.edu; 12 Department of Nursing and Dental Medicine, Faculty of Medicine, Lucian Blaga University of Sibiu, 550024 Sibiu, Romania; cosmina.diaconu@ulbsibiu.ro; 13 Clinic of Physical Medicine and Rehabilitation, County Clinical Emergency Hospital Sibiu, Bulevardul Corneliu Coposu 2–4, 550245 Sibiu, Romania

**Keywords:** leptin, asthma, allergic rhinitis, non-allergic asthma, immunometabolism, adipokines, biomarkers, disease severity

## Abstract

**Background/Objectives**: Leptin is a pleiotropic adipokine linking energy metabolism with innate and adaptive immunity. Its relationship with clinical severity across distinct airway disease phenotypes remains insufficiently characterized. This study examined serum leptin concentrations in adults with allergic rhinitis, non-allergic asthma, and allergic asthma associated with allergic rhinitis. **Methods**: This single-center retrospective observational study analyzed fully anonymized clinical and laboratory data from 88 adults: patients with allergic rhinitis *(n* = 31), non-allergic asthma (n = 15), or allergic asthma with allergic rhinitis (n = 22) and healthy controls (n = 20). Serum leptin was measured using a quantitative sandwich enzyme-linked immunosorbent assay. Disease severity was classified using the Allergic Rhinitis and its Impact on Asthma and Global Initiative for Asthma frameworks applicable during the study period. Statistical analyses were performed using IBM SPSS Statistics, version 26.0 (IBM Corp., Armonk, NY, USA), and available original statistical outputs were summarized using retained t statistics and two-sided *p* values. **Results**: Stage-specific mean serum leptin concentrations were heterogeneous and non-monotonic. Statistically significant relationships between leptin concentration and disease stage were reported for allergic rhinitis (t = 2.844; *p* = 0.008), non-allergic asthma (t = 4.240; *p* = 0.001), and allergic asthma with allergic rhinitis (t = 2.700; *p* = 0.013). In the allergic rhinitis subgroup, 64.5% of participants had normal weight, 32.3% were overweight, and 3.2% had grade II obesity; weight category was not significantly related to rhinitis stage. **Conclusions**: The retained analyses indicate statistically significant relationships between serum leptin concentration and clinical disease stage across the investigated airway phenotypes. However, the stage-specific descriptive means were heterogeneous and non-monotonic and therefore do not support a uniform progressive increase in leptin with increasing disease severity. Because complete model outputs and covariate-adjusted estimates were unavailable, these findings should be regarded as exploratory and require prospective validation with appropriate adjustment for adiposity and other relevant metabolic confounders.

## 1. Introduction

Airway diseases, particularly allergic rhinitis and asthma, are among the most common chronic inflammatory disorders worldwide and continue to impose a substantial healthcare burden [1,2,3,4]. Although airway inflammation remains their defining pathological feature, increasing evidence indicates that systemic metabolic dysregulation also contributes to disease severity and persistence. This evolving concept, known as immunometabolism, recognizes the dynamic interaction between metabolic pathways and immune responses in chronic inflammatory diseases [5,6,7,8].

Among adipokines, leptin has attracted particular attention because of its dual metabolic and immunological functions [9,10,11,12,13]. Besides regulating appetite and energy homeostasis through hypothalamic signaling, leptin exerts potent immunomodulatory and pro-inflammatory effects by promoting macrophage activation, cytokine production, T-cell proliferation, and polarization toward Th1 and Th17 immune responses [9,10,11,12,13]. Increasing evidence also suggests interactions between leptin signaling and eosinophilic airway inflammation, airway remodeling, and bronchial hyperresponsiveness [11,12,13,14,15,16,17,18,19].

Previous studies have associated elevated circulating leptin concentrations with obesity-related asthma, impaired lung function, and increased disease severity [14,15,16,17,18,19,20]. However, available evidence remains heterogeneous, and direct comparisons across different airway disease phenotypes are scarce. In particular, whether leptin exhibits similar biological behavior in allergic rhinitis, non-allergic asthma, and combined upper and lower airway disease remains insufficiently understood [19,20,21]. A better understanding of these relationships may provide further insight into the immunometabolic mechanisms underlying chronic airway inflammation and facilitate future biomarker-based patient stratification [3,22,23,24,25,26,27,28,29,30].

Therefore, the aim of this study was to describe serum leptin concentrations across clinical disease stages in patients with allergic rhinitis, non-allergic asthma, and allergic asthma associated with allergic rhinitis and to examine the reported relationship between circulating leptin levels and disease stage within these phenotypes.

## 2. Materials and Methods

### 2.1. Study Design

**Figure 1:**
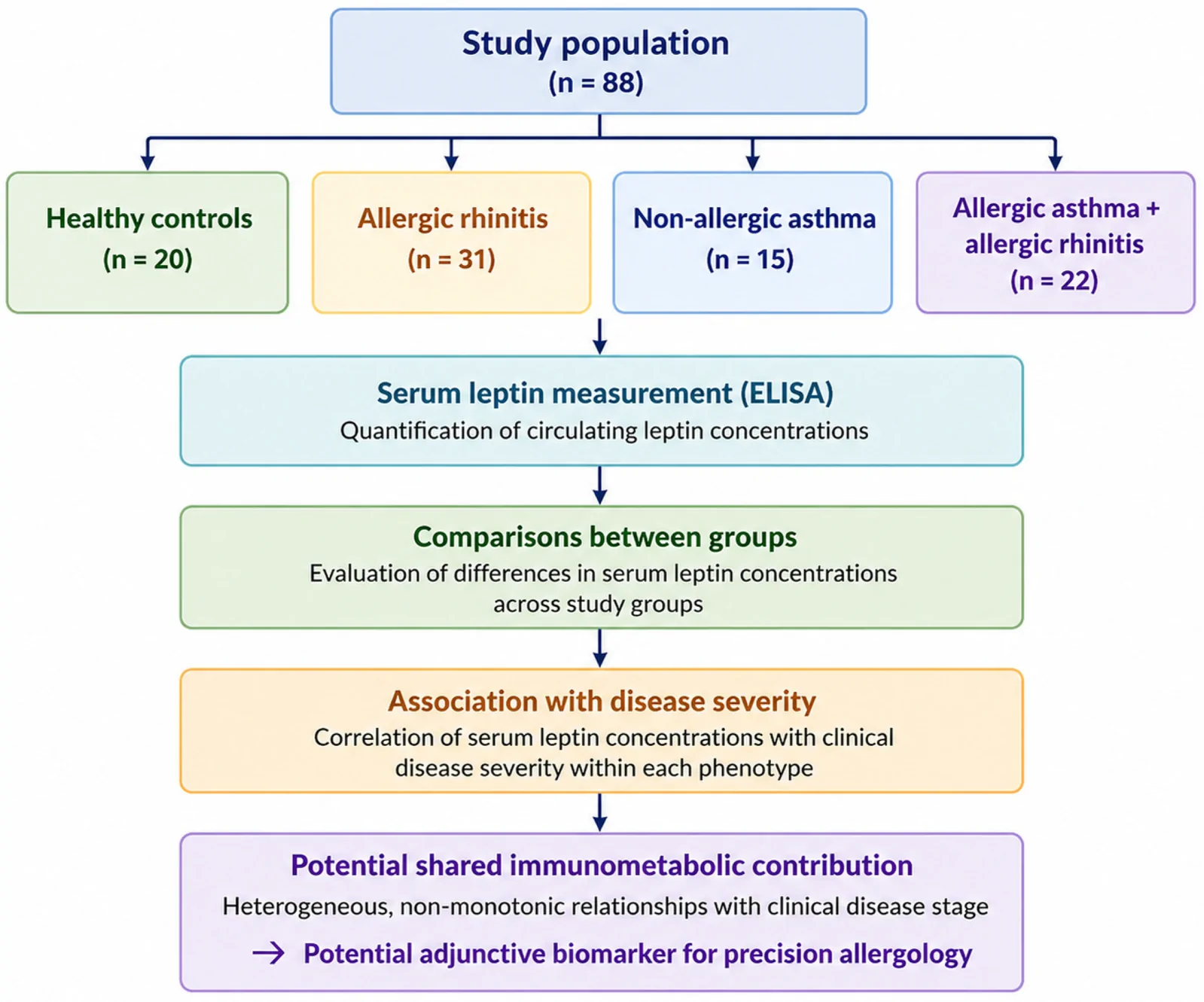
Study design and analytical workflow. Overview of participant stratification, serum leptin measurement, comparisons among groups, and assessment of the association with disease severity. Colors are used solely to visually distinguish the different stages of the analytical workflow and do not represent additional variables or statistical categories.

This single-center retrospective observational comparative study evaluated existing, fully anonymized clinical and laboratory data from adults with different airway disease phenotypes and healthy controls. The study was conducted at the Department of Allergy, County Clinical Emergency Hospital Sibiu, Romania. The primary analysis focused on stage-specific serum leptin concentrations and their reported relationship with disease stage within the three clinical phenotypes. The study design and analytical workflow are summarized in Figure 1.

### 2.2. Participants and Recruitment

A total of 88 adults were included in the retrospective analysis. Participants were recruited between January and December 2025. They were not newly diagnosed at the time of inclusion, as eligibility required a documented disease duration of at least two years. The retrospective archived dataset did not contain sufficiently complete information to reconstruct individual maintenance treatment, recent treatment changes, or the exact timing of blood sampling in relation to treatment initiation. These variables were therefore not included in the present analysis and are acknowledged as potential sources of residual confounding.

The study population comprised 31 adults with allergic rhinitis, 15 adults with non-allergic asthma, 22 adults with allergic asthma associated with allergic rhinitis, and 20 healthy adult control subjects.

Participants were classified according to their established clinical diagnoses recorded in the source documentation.

All patients underwent standardized clinical evaluation, and the diagnosis of allergic rhinitis and asthma was established according to internationally accepted diagnostic criteria applicable during the study period [1,2].

### 2.3. Inclusion and Exclusion Criteria

Adults aged 18 years or older were eligible if the available records documented a confirmed diagnosis of allergic rhinitis or asthma, a disease duration of at least two years, and fulfillment of the disease-specific diagnostic criteria. Pregnant women, patients with severe chronic systemic diseases, and subjects with alternative causes of rhinitis or asthma were excluded. Healthy adult controls had no documented history of allergic, autoimmune, or chronic inflammatory diseases.

### 2.4. Clinical Assessment

Demographic and clinical characteristics, including BMI and obesity status when available, smoking status, family history of allergic diseases, disease duration, disease severity, sensitization profile, associated allergic conjunctivitis, season of birth, and place of residence, were extracted from the available clinical records.

### 2.5. Assessment of Disease Severity

Allergic rhinitis severity was classified according to the ARIA framework. For the purposes of the present analysis, four clinical categories were used: Stage 1, intermittent mild allergic rhinitis; Stage 2, intermittent moderate/severe allergic rhinitis; Stage 3, persistent mild allergic rhinitis; and Stage 4, persistent moderate/severe allergic rhinitis.

For asthma, the four severity categories recorded in the source documentation were used in the original analysis: Stage 1, intermittent asthma; Stage 2, mild persistent asthma; Stage 3, moderate persistent asthma; and Stage 4, severe persistent asthma. Among the 15 patients with non-allergic asthma, 3 were classified as having intermittent asthma, 3 as mild persistent asthma, 4 as moderate persistent asthma, and 5 as severe persistent asthma. These numerical stage labels reflect the clinical severity categories recorded in the original dataset and should not be interpreted as equivalent to current GINA treatment-step categories [1,2].

Because sufficiently complete maintenance-treatment data were unavailable in the archived dataset, asthma severity could not be retrospectively reclassified according to the intensity of treatment required to achieve or maintain disease control. The present analysis therefore retained the clinical severity categories recorded in the original source documentation.

### 2.6. Measurement of Serum Leptin

Serum leptin concentrations were measured using a commercially available quantitative sandwich enzyme-linked immunosorbent assay (ELISA) (Quantikine^®^, R&D Systems, Minneapolis, MN, USA), according to the manufacturer’s instructions. Briefly, serum samples were incubated in microplates coated with monoclonal anti-human leptin antibodies. Following washing procedures, enzyme-conjugated secondary antibodies and chromogenic substrate were added. Optical density was measured spectrophotometrically, and leptin concentrations were calculated from a standard calibration curve [10].

### 2.7. Statistical Analysis

Statistical analyses were performed using IBM SPSS Statistics, version 26.0 (IBM Corp., Armonk, NY, USA).Continuous variables were summarized as mean ± standard deviation or median (interquartile range), according to their distribution, and categorical variables as frequencies and percentages. The relationship between serum leptin concentration and clinical disease stage was assessed separately within each phenotype in the original analysis. The retained output comprised a t statistic and corresponding two-sided *p* value for each phenotype. Because the complete archived SPSS output was unavailable for the present retrospective manuscript, the exact model specification, regression coefficients, confidence intervals, model-fit indices, and covariate-adjusted estimates could not be reconstructed. The reported findings were therefore interpreted as exploratory, and *p* < 0.05 was considered statistically significant.

Because of the limited sample size within phenotype groups, all analyses were considered exploratory and the findings were interpreted cautiously.

## 3. Results

### 3.1. Study Population

A total of 88 adults were included in this retrospective comparative observational study: 31 with allergic rhinitis, 15 with non-allergic asthma, 22 with allergic asthma associated with allergic rhinitis, and 20 healthy adult controls.

The demographic and clinical characteristics of the study population are summarized in Table 1. Patients with allergic rhinitis showed a slight male predominance, whereas the non-allergic asthma and allergic asthma associated with allergic rhinitis groups exhibited a more balanced sex distribution. Current smoking was infrequent across all study groups, and approximately one-third of patients in each disease group reported a positive family history of allergic disease. In the allergic rhinitis subgroup, 20 patients (64.5%) had normal weight, 10 (32.3%) were overweight, and one (3.2%) had grade II obesity. No significant relationship between weight category and allergic rhinitis stage was reported in the original subgroup analysis.

Clinical severity varied across all disease phenotypes, thereby providing an appropriate framework for evaluating the relationship between serum leptin concentrations and disease severity. Allergic conjunctivitis was frequently observed in patients with allergic rhinitis, whereas nasal polyposis was identified only in a small proportion of patients with non-allergic asthma.

### 3.2. Association Between Serum Leptin Concentrations and Disease Severity

**Figure 2:**
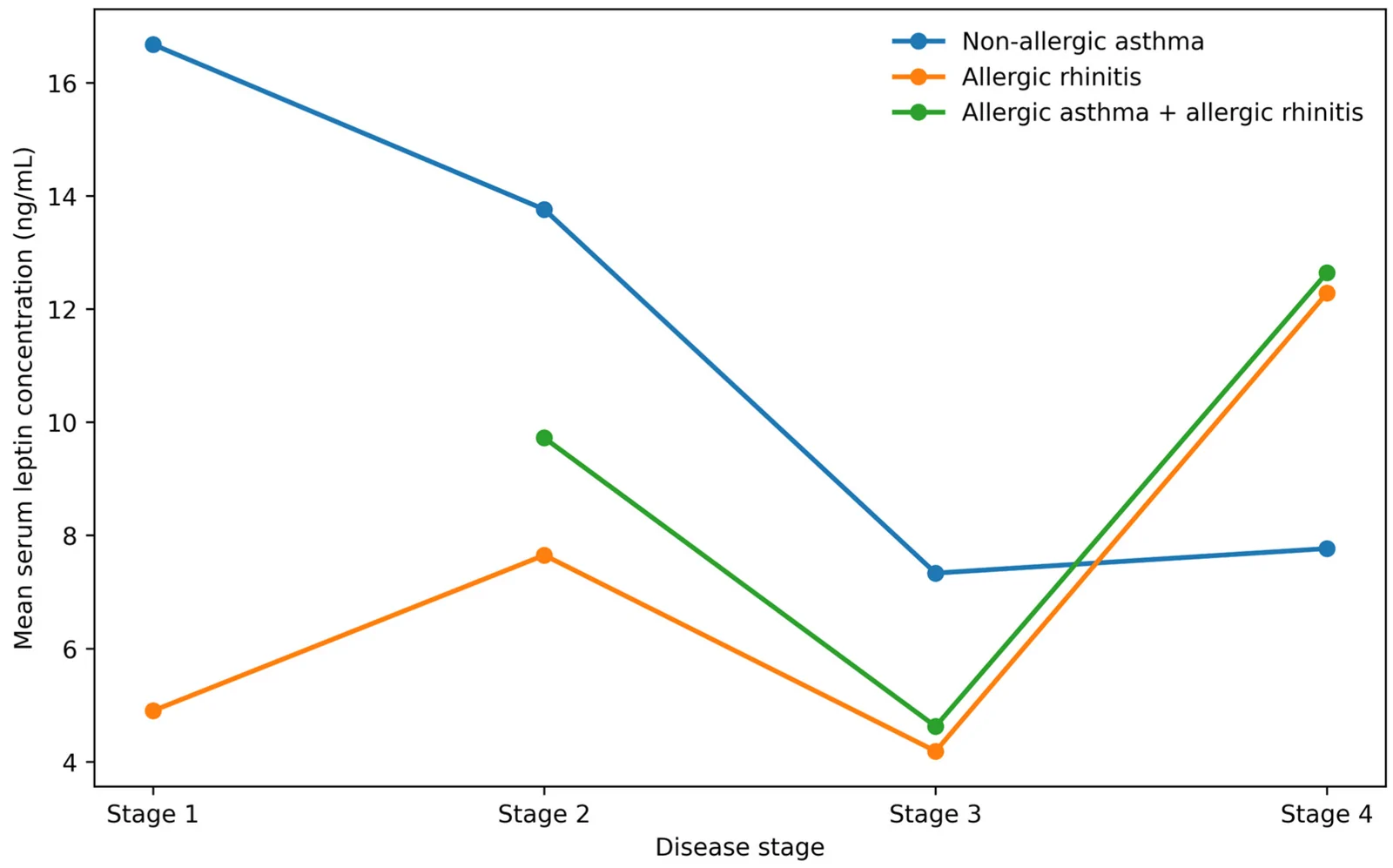
Arithmetic mean serum leptin concentrations (ng/mL) according to disease stage. The graph displays descriptive means only; stage-specific sample sizes and dispersion measures were unavailable.

Stage-specific arithmetic mean serum leptin concentrations are presented in Table 2 and Figure 2. The descriptive values were not strictly monotonic within every phenotype. The retained statistical analyses nevertheless revealed a statistically significant relationship between leptin concentration and disease stage in each disease group.

In allergic rhinitis, mean leptin concentrations across stages 1–4 were 4.90, 7.65, 4.19, and 12.28 ng/mL, respectively. The highest mean value was observed in stage 4. The retained analysis indicated a statistically significant relationship between leptin concentration and rhinitis stage (t = 2.844; *p* = 0.008).

In non-allergic asthma, mean leptin concentrations across stages 1–4 were 16.68, 13.76, 7.33, and 7.77 ng/mL, respectively. The descriptive pattern was predominantly decreasing across severity categories and therefore did not demonstrate a progressive increase in leptin with increasing disease stage. Nevertheless, the retained original statistical output showed a statistically significant relationship between leptin concentration and disease stage (t = 4.240; *p* = 0.001). Because the complete archived statistical model, including the model specification, effect estimates, and confidence intervals, was unavailable, the direction and functional form of this relationship cannot be established from the retained t statistic and *p* value alone.

In allergic asthma associated with allergic rhinitis, no stage-1 mean was reported; mean leptin concentrations for stages 2–4 were 9.72, 4.63, and 12.64 ng/mL, respectively. The highest mean was observed in stage 4, and the retained analysis revealed a statistically significant relationship with disease stage (t = 2.700; *p* = 0.013).

The available statistical associations between serum leptin concentration and disease severity are summarized in Table 3.

The retained original analyses revealed statistically significant relationships between serum leptin concentration and disease stage in all three phenotypes. However, the stage-specific descriptive means were heterogeneous and non-monotonic and therefore should not be interpreted as demonstrating a progressive or linear increase in leptin with increasing disease severity. This distinction is particularly important for non-allergic asthma, in which the descriptive means showed a predominantly decreasing pattern across severity categories. Because the complete original statistical models, stage-specific sample sizes, dispersion measures, and covariate-adjusted estimates were unavailable, these inferential findings should be considered exploratory and do not establish the direction or functional form of the relationships.

**Figure 3:**
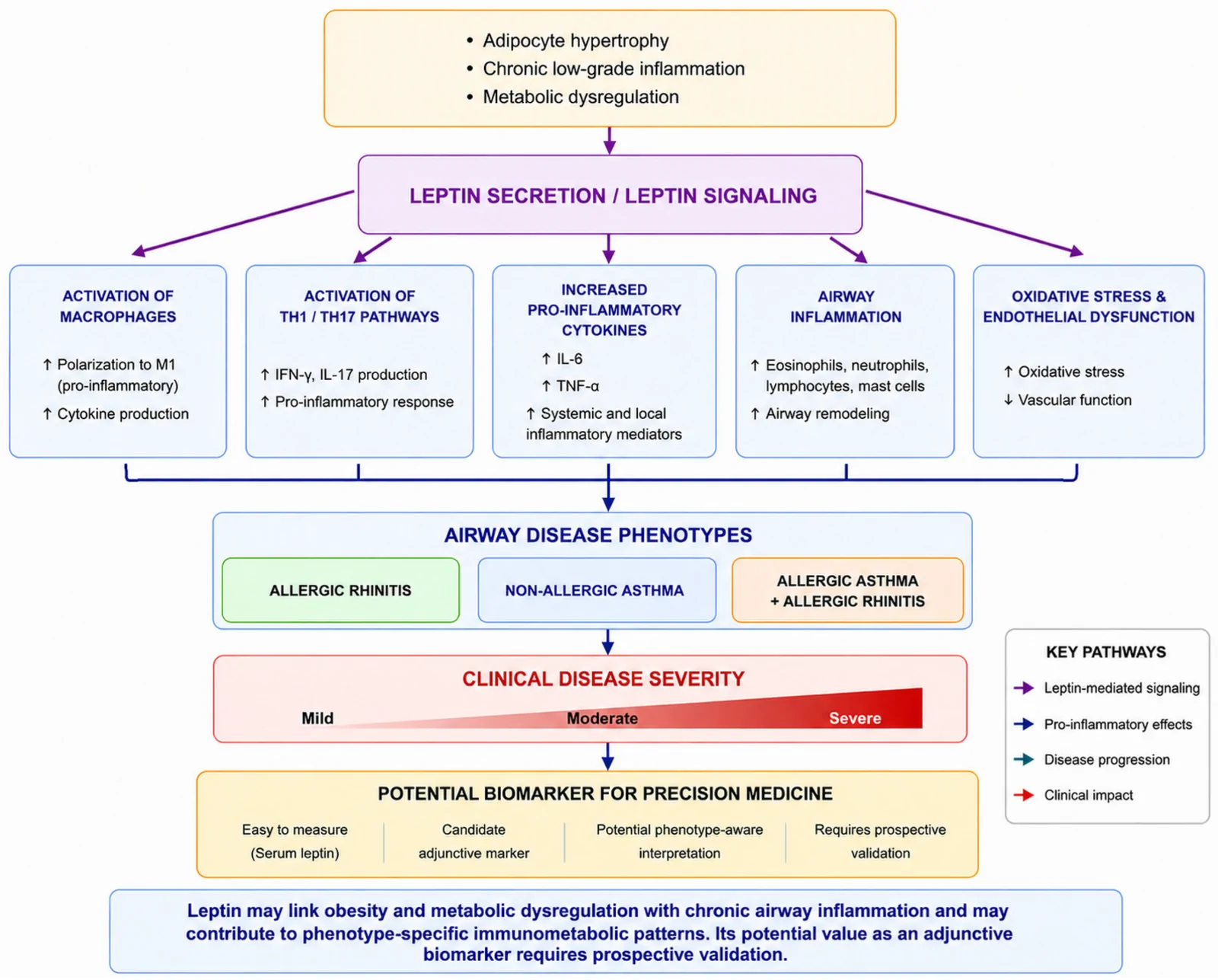
Proposed immunometabolic framework linking leptin signaling with airway inflammation and disease severity. Arrows indicate the direction of the proposed biological pathways. Colors and symbols correspond to the key pathway categories shown in the figure legend, including leptin-mediated signaling, pro-inflammatory effects, disease progression, and clinical impact. This conceptual diagram integrates the present observations with existing immunometabolic mechanisms.

A schematic representation of the proposed biological framework integrating these findings with current concepts of immunometabolism is presented in Figure 3.

## 4. Discussion

### 4.1. Principal Findings

The retained analyses showed statistically significant relationships between serum leptin concentration and disease stage across allergic rhinitis, non-allergic asthma, and allergic asthma associated with allergic rhinitis. However, the stage-specific descriptive means were heterogeneous and non-monotonic and therefore do not demonstrate a linear dose–response relationship between leptin and increasing disease severity. This distinction was particularly evident in non-allergic asthma, where the descriptive pattern was predominantly decreasing across severity categories.

Because the complete original statistical models, stage-specific sample sizes, dispersion measures, and covariate-adjusted estimates were unavailable, the direction, magnitude, and independence of these relationships cannot be established with confidence. The findings should therefore be considered exploratory and hypothesis-generating and require prospective validation using complete individual-level data and adjustment for adiposity and other relevant confounders.

The biological plausibility of these findings is supported by the established immunometabolic role of leptin. Beyond its function in energy homeostasis, leptin modulates innate and adaptive immunity and may promote macrophage activation, pro-inflammatory cytokine production, and Th1/Th17 responses [9,10,11,12,13]. Nevertheless, the present observational findings do not establish causality, and the heterogeneous leptin patterns indicate that its relationship with airway disease severity is likely influenced by multiple metabolic and inflammatory factors.

### 4.2. Leptin and Chronic Airway Inflammation: Bridging Metabolism and Immunity

Airway diseases involve interactions between local inflammation and systemic metabolic pathways. Adipose tissue functions as an immunoendocrine organ, and leptin may influence macrophage activation, eosinophil survival, T-cell responses, and pro-inflammatory cytokine production [31,32,33]. These mechanisms provide biological plausibility for a relationship between leptin and airway inflammation. However, the heterogeneous and non-monotonic stage-specific concentrations observed in the present study indicate that leptin is unlikely to reflect disease severity through a simple dose–response relationship. Its potential role should therefore be considered within a broader network of inflammatory, metabolic, environmental, and clinical factors.

### 4.3. Leptin and Disease Severity: Clinical Implications of the Present Findings

A clinically relevant finding was that the retained analyses revealed statistically significant relationships between serum leptin concentration and disease stage in each investigated phenotype. However, the stage-specific arithmetic means were heterogeneous and not uniformly monotonic, so the results do not demonstrate a progressive increase in leptin with increasing severity.

The non-allergic asthma group yielded the largest retained t statistic and the smallest *p* value. This may be compatible with an important contribution of systemic metabolic and innate immune mechanisms in non-allergic asthma, but the direction, magnitude, and independence of the association cannot be established without the complete statistical model and adjustment for BMI, age, sex, and other confounders.

The contrasting descriptive patterns observed between allergic and non-allergic phenotypes are of potential interest. However, the clinical phenotype of non-allergic asthma should not be considered synonymous with a non-Type 2 inflammatory endotype. Because specific endotype-defining biomarkers were not systematically available in the archived dataset, the present findings cannot establish whether the observed differences reflect Type 2 versus non-Type 2 inflammatory mechanisms.

Across the allergic phenotypes, the heterogeneous stage-specific patterns indicate that the relationship between circulating leptin and clinical severity is unlikely to be explained by a simple linear dose–response model. Differences in adiposity, treatment exposure, inflammatory endotype, sex, and other metabolic factors may contribute to this variability and should be addressed in future adequately powered, covariate-adjusted studies.

From a clinical perspective, serum leptin cannot currently be considered a standalone diagnostic or severity biomarker. The present exploratory findings support further investigation of leptin within multimarker approaches that also incorporate adiposity, conventional inflammatory biomarkers, pulmonary function, and clinical phenotype.

### 4.4. Leptin as a Potential Biomarker for Precision Allergology

Precision medicine in airway disease increasingly relies on clinical phenotypes, molecular endotypes, and biomarker combinations rather than single markers [23,24,25,26,27,28,29,30,34,35,36,37,38]. In this context, serum leptin may warrant further investigation as a component of multimarker approaches integrating adiposity, inflammatory biomarkers, pulmonary function, and clinical phenotype. However, the present data do not support its use as a standalone diagnostic or severity biomarker. Moreover, leptin alone may not adequately characterize adipose-tissue dysfunction. Adiponectin and leptin provide complementary metabolic information, and the adiponectin/leptin ratio has been proposed as an indicator of dysfunctional adipose tissue and obesity-associated cardiometabolic risk [39]. Although adiponectin was not measured in the present study, future airway-disease studies should consider simultaneous adipokine profiling rather than assessment of leptin in isolation. Prospective studies with appropriate adjustment for BMI, sex, age, smoking status, treatment, and metabolic factors are required to determine its independent clinical value.

### 4.5. Strengths of the Study

The main strength of this study is the evaluation of serum leptin across three clinically distinct airway disease phenotypes within the same study population. In addition, the analysis focused on disease-stage relationships rather than solely on comparisons with healthy controls. This phenotype-based approach provides exploratory information relevant to the potential immunometabolic role of leptin and supports the design of future studies using more comprehensive biomarker and metabolic profiling.

### 4.6. Limitations

Several limitations should be acknowledged. This was a single-center retrospective observational study with a relatively small sample size, limiting generalizability and precluding causal or temporal inferences. Complete individual-level statistical outputs, stage-specific sample sizes and dispersion measures, and covariate-adjusted estimates were unavailable. Detailed BMI and weight-category data were not available for all groups, limiting adjustment for adiposity, an important determinant of circulating leptin. Detailed metabolic comorbidities, including diabetes status and markers of insulin resistance, were not systematically available for all participants. Consequently, the potential influence of metabolic dysfunction or leptin resistance on circulating leptin concentrations could not be assessed. Information on maintenance treatment, recent treatment changes, and recent or long-term systemic corticosteroid exposure could not be completely reconstructed, representing a potential source of residual confounding. In addition, other adipokines and inflammatory mediators, including adiponectin, resistin, IL-6, TNF-α, and IL-17, were not measured simultaneously. These limitations require cautious interpretation of the reported associations.

### 4.7. Future Perspectives

Future prospective multicenter studies should evaluate serum leptin using complete individual-level data, larger phenotype-specific cohorts, and adjustment for adiposity, treatment, and metabolic confounders. Combining leptin with other adipokines, established inflammatory biomarkers, pulmonary-function measures, and molecular profiling may clarify whether it contributes meaningfully to immunometabolic endotyping or patient stratification in airway disease.

## 5. Conclusions

The retained analyses indicate statistically significant relationships between serum leptin concentration and clinical disease stage across the investigated airway phenotypes. However, the stage-specific descriptive means were heterogeneous and non-monotonic and therefore do not support a uniform progressive increase in leptin with increasing disease severity. In particular, the descriptive pattern in non-allergic asthma was predominantly decreasing across severity categories. Because stage-specific sample sizes and dispersion measures, complete archived statistical model outputs, and covariate-adjusted estimates were unavailable, neither the direction nor the functional form of the reported relationships can be established with confidence. The findings should therefore be regarded as exploratory and hypothesis-generating. Prospective multicenter studies using complete individual-level data and appropriate adjustment for BMI, age, sex, smoking status, treatment, and other relevant metabolic confounders are needed to determine whether serum leptin has value as an adjunctive biomarker in airway disease.

## Figures and Tables

**Table 1:** Baseline demographic and clinical characteristics of the study population.

Characteristic	Allergic Rhinitis (*n* = 31)	Non-Allergic Asthma (*n* = 15)	Allergic Asthma + Allergic Rhinitis (*n* = 22)	Healthy Controls (*n* = 20)
Female sex, *n* (%)	14 (45.2)	7 (46.7)	12 (54.5)	17 (85.0)
Male sex, *n* (%)	17 (54.8)	8 (53.3)	10 (45.5)	3 (15.0)
Current smokers, n (%)	4 (12.9)	1 (6.7)	0 (0.0)	0 (0.0)
Positive family history of allergic disease, *n* (%)	10 (32.3)	5 (33.3)	7 (31.8)	0 (0.0)
Allergic conjunctivitis, n (%)	17 (54.8)	NA	9 (40.9)	NA
Nasal polyposis, *n* (%)	NA	1 (6.7)	NA	NA

Data are presented as number (%). NA, not applicable.

**Table 2:** Arithmetic mean serum leptin concentrations (ng/mL) according to disease stage.

Disease Stage	Non-Allergic Asthma, Mean Leptin (ng/mL)	Allergic Rhinitis, Mean Leptin (ng/mL)	Allergic Asthma + Allergic Rhinitis, Mean Leptin (ng/mL)
Stage 1	16.675	4.900	NR
Stage 2	13.760	7.650	9.7222
Stage 3	7.3333	4.1864	4.625
Stage 4	7.7667	12.280	12.640

Values are arithmetic means derived from the original analysis. NR, not reported. Stage-specific sample sizes and measures of dispersion were unavailable; therefore, these descriptive values should be interpreted together with the reported exploratory inferential results.

**Table 3:** Available statistical associations between serum leptin concentration and disease severity.

Phenotype	t Statistic	*p* Value	Interpretation
Allergic rhinitis	2.844	0.008	Statistically significant relationship
Non-allergic asthma	4.240	0.001	Largest retained t statistic
Allergic asthma + allergic rhinitis	2.700	0.013	Statistically significant relationship

## Data Availability

The data that support the findings of this study are available from the corresponding author upon reasonable request, subject to ethical and privacy restrictions.
